# CT-Guided Percutaneous Microwave Ablation of Sclerosing Hepatic Carcinoma

**DOI:** 10.1155/2020/8881978

**Published:** 2020-07-15

**Authors:** Hongshen Song, Huaiyin Ding, Chuandong Zhu

**Affiliations:** ^1^Department of Radiology, The Second Hospital of Nanjing, Nanjing University of Chinese Medicine, Nanjing 210003, China; ^2^Department of Ultrasound, The Second Hospital of Nanjing, Nanjing University of Chinese Medicine, Nanjing 210003, China; ^3^Department of Pathology, The Second Hospital of Nanjing, Nanjing University of Chinese Medicine, Nanjing 210003, China; ^4^Department of Oncology, The Second Hospital of Nanjing, Nanjing University of Chinese Medicine, Nanjing 210003, China; ^5^Liver Cancer Treatment Center, The Second Hospital of Nanjing, Nanjing University of Chinese Medicine, Nanjing 210003, China

## Abstract

Sclerosing hepatic carcinoma (SHC) is a rare subtype of hepatic carcinoma that can be caused by various pathogeneses. The histological characteristics of SHC demonstrate its high resistance to chemoembolization and thermal ablation; thus, surgical resection represents the primary option for the majority of patients. However, a small proportion of patients who cannot withstand surgery or who have inoperable tumors may not receive adequate treatment, causing the progression of cancer and related high mortality. To overcome the high puncture resistance, high thermal resistance, and poor thermal conductivity of microwave ablation, we developed percutaneous no-touch multiple-site microwave ablation (NTMSWA) to ablate SHC lesions. In this retrospective study, 96 and 41 patients underwent NTMSWA and surgery, respectively. In the NTMSWA group, tumor size and histological classification were determined by medical imaging and tissue biopsy before ablation, and then a personalized ablation regimen was performed. Complete ablation was achieved in a single session in 81 out of 96 (84.4%) patients. The median survival (MS) of the 90 patients who underwent NTMSWA was 51 months, and the overall survival (OS) rate at 5 years was 49.1%. In contrast, the MS in the control group was 57 months, and the OS rate at 5 years was 56.3%. There was no significant difference between the two groups, indicating that SHC <50 mm in size can be effectively ablated with NTMSWA. By adopting no-touch, multiple-site, low-power, intermittent ablation, SHC less than 50 mm in size can be completely ablated.

## 1. Introduction

**Figure 1:**
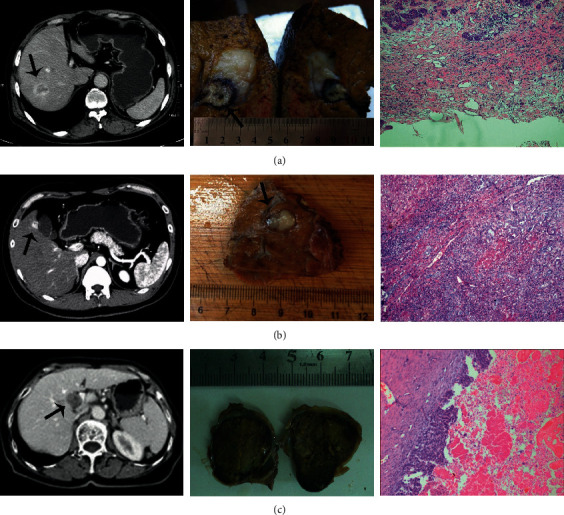
Three cases of ablation failure experienced before 2012 when preoperative biopsy was not routinely performed. (a) A 64-year-old male with sclerosing hepatocellular carcinoma who underwent surgical resection after ablation failure. The initial ablation site was indicated, showing the poor ablation scope and efficacy. The subsequent immunohistostaining revealed a severe fibrous component (∼80% of the tumor tissue) causing unexpected hardness, a high level of heat resistance, and poor heat conduction. (b) A 37-year-old male patient with bile duct adenocarcinoma who underwent surgical resection. The immunohistostaining indicated mild fibrosis (∼40%). The failure cause was the displacement of the rubbery tumor during puncture and a high level of heat resistance. (c) A 72-year-old female patient with intrahepatic cholangiocarcinoma. Typical cystic changes were found in histological sections. The rigid tumor capsule composed of a fibrous component and necrotic tumor tissue completely prevented puncture.

Sclerosing hepatic carcinoma (SHC) is a subtype of hepatic carcinoma (HC), accounting for less than 5% of total HC cases. It can be caused by the increased proliferation of fibroblasts/myofibroblasts and fibrosis in its tumor microenvironment [1]. Tumor fibrosis can be further classified into three major patterns: (i) fibrosis extending along the sinusoid-like blood spaces with atrophy of the tumor trabeculae, (ii) dense fibrosis with hyalinization separating the tumor into tumor nests with various sizes, and (iii) fibrosis with a lamellar pattern. These subtypes of fibrosis often coexist to various degrees [2]. Therefore, SHC has a variety of pathological morphologies, but it is normally characterized by fibrous stroma in which neoplastic tumor structures are embedded, forming cords or trabeculae without typical necrosis or cystic changes [3]. Surgical resection is the major option in the treatment of SHC [4]. However, patients who cannot withstand surgery may have to seek other therapeutics, such as conventional chemotherapy, transcatheter arterial embolization, and thermal ablation [4]. Of note, SHC is particularly difficult to treat with chemotherapy and embolization because of its inherent resistance to chemotherapy, fibrosis, and relatively poor blood supply [4]. In comparison, thermal ablation has gained wide acceptance. In microwave ablation (MWA), the electromagnetic waves in the microwave energy spectrum can be used to produce tissue-heating effects, thereby killing nearby cells [5]. Minimally invasive surgery is the preferred approach for a growing number of major surgeries that previously involved large incisions and a lengthy recuperation period [6]. Hence, image-guided percutaneous MWA has been widely used to ablate solid tumor lesions in the lung, liver and other organs. However, a few challenges must be overcome before applying MWA for the treatment of SHC. Based on failure experience (Figure 1), we noticed that fibrous components and/or extensive necrotic tumor tissue can endow the lesion with high puncture resistance, high thermal resistance, and poor thermal conductivity, causing frequent ablation failure and a high recurrence rate. To improve the therapeutic efficacy, we developed no-touch multiple-site ablation (NTMSWA) and optimized the relevant parameters for personalized ablation. In brief, tissue biopsy must be performed before ablation. On the basis of immunohistochemistry and tumor size, we can optimize percutaneous CT-guided NTMSWA by selecting multiple ablation sites around the tumor. Furthermore, microwave power, ablation time, and intervals will be optimized to ablate tumors. This method achieved complete ablation in a single session in 84.4% of patients. Compared to surgery-treated patients, in these patients, no significant difference was found in median survival (MS), overall survival (OS), or local tumor progression (LTP), indicating that this method can efficiently ablate SHC less than 50 mm in size with minimal invasiveness.

## 2. Materials and Methods

### 2.1. Patients

This study was approved by the Institutional Review Board of The Second Hospital of Nanjing. It abided the guidelines of the Helsinki Declaration. Written informed consent was obtained from all patients. From September 2013 to August 2015, 96 and 41 patients were treated with NTMSWA and surgery, respectively. The enrollment criteria were as follows: (1) biopsy-proven SHC; (2) single nodules, with a size ≤50 mm; (3) without extrahepatic metastases; (4) without obstinate malignant ascites or portal vein tumor thrombus; (5) Child-Pugh class A or B; and (6) prothrombin time ratio >50% [7]. The exclusion criteria were (1) absent or poor visualization of the nodule on CT and (2) with contraindications of MWA [7].

### 2.2. Optimization of Microwave Power and Ablation Time with Porcine Liver In Vitro

The freshly excised porcine liver was bought from a slaughterhouse and recovered to 37°C before MWA. The microwave power ranged from 30 to 50 Watts, and the ablation time varied from 3 to 8 min. The average depth of puncture was 4–7 cm. To measure the ablation temperature generated by the microwave, the thermocouples were placed at 5-, 10-, 15-, and 20-mm positions of the electrode. Ablation at a fixed microwave power and ablation time was repeated five times. Then, ablation parameters, including morphology, ablation size, and carbonization size, were measured.

### 2.3. Treatment, Follow-Up, and Statistical Analysis

**Figure 2:**
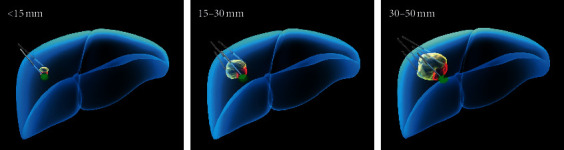
Depending on the biopsy and tumor size, multiple-site ablation would be performed to destroy tumors.

The 18 G electrode and ECO-100C microwave generator (2450 MHz with wavelength of 12.25 cm) were used for ablation under CT guidance. Ablation was performed at 30–50 Watts with a desired temperature of 95°C for 6 to 20 min. Depending on the histologic examination and tumor size, respective ablation was performed (Figure 2). Briefly, the probe penetrated tumors <10 mm in size for direct ablation; alternatively, 2 to 5 sites of NTMSWA were performed to destroy tumors <15 mm, 15–30 mm, and 30–50 mm in size. In the control group, liver resection was performed. Through the J-shaped incision, an ultrasonic aspirator was used to excise the tumor followed by suturing. To assess the therapeutic efficacy, all patients underwent contrast-enhanced CT 1 month after treatment. Complete ablation was defined as complete nonenhancement of the treated lesion. Additional ablation was performed to ablate the residual tumor. NTMSWA failure was considered if the tumor remained viable after the second ablation. In the control group, biopsy was not purposely performed before surgery. As usual, surgical excision was directly performed if patients with operable tumors could withstand surgery. Patients were allocated to the control group if SHC was identified by the histopathological examination of postsurgical liver specimens. Resection was performed for Child A patients and for highly selected Child B patients with small tumors in whom liver transplantation was not possible. To make the data comparable to the radiofrequency group, tumor staging was defined based on the preoperative assessment with imaging techniques. Pathology of all specimens confirmed the diagnosis in all cases. All resections were considered radical (tumor-free resection margins confirmed by pathology); in detail, 9 wedge resections, 17 segmentectomies, 4 bisegmentectomies, and 11 major resections (3 or more hepatic segments resected) were performed. Significant differences (*p* < 0.05) were determined using an independent student's *t*-test or chi-square test. The LTP and OS were determined with the Kaplan–Meier method and compared with the log-rank test.

## 3. Results

### 3.1. Ablation Range in a Porcine Liver

**Figure 3:**
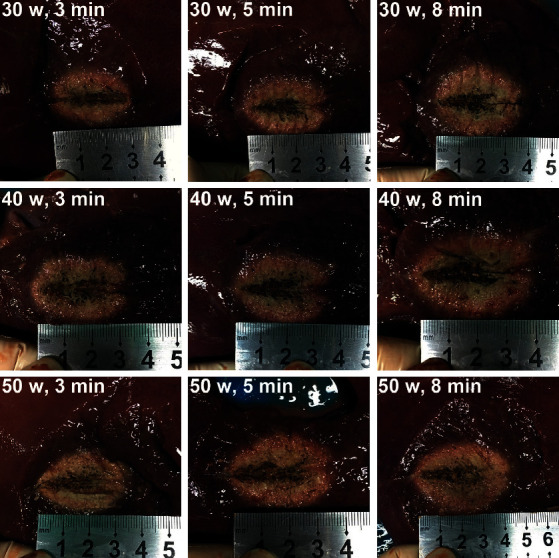
Ablation range of the microwave probe at varying power and time using a freshly excised porcine liver.

We first scrutinized the ablation range of the MWA probe using a freshly excised porcine liver. The morphology of the ablated porcine liver showed a typical carbonization zone, an area of coagulation necrosis, an area of inflammation and hemorrhage or incomplete cell death, and a normal liver parenchyma (Figure 3). In general, the average ablation scope increased as the ablation power/time increased (Table 1). The investigation could assist us in determining the ideal ablation power, sites, and time while reducing normal tissue damage and improving the therapeutic ratio.

### 3.2. Tumor Response, Complications, and Follow-Up

**Figure 4:**
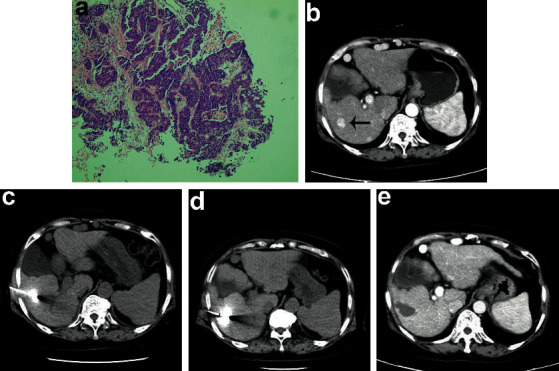
A 67-year-old male patient with bile duct adenocarcinoma (size: 14 × 11 mm) underwent two-site MWA with a single probe. (a)The histological section (×100 objective lens) shows ∼50% fibrous components. (b–e) CT images show the lesion before and after no-touch ablation at two opposite sites.

Before NTMSWA, all 96 patients underwent tissue biopsy to identify the histological classification, especially the degree of fibrosis and necrosis (Table 2). The tumor volume was determined by either CT or MRI. On the basis of the histologic examination and tumor size, the personalized ablation regimens were judiciously contrived. In brief, depending on the tumor size, up to 5 sites abutting the tumor margin were selected for ablation. Moreover, a power of up to 50 Watts was selected to intermittently ablate tumors according to the histologic features. The average ablation time was 12 ± 2.5 min (ranging from 6 to 20 min) with intervals of 30 to 60 seconds. Moreover, a wide safety margin (>10 mm) was attained. For instance, a 67-year-old male patient with bile duct adenocarcinoma with a tumor size of 14 × 11 mm underwent two-site ablation with a single probe (Figure 4), and CT images showed no viable residual tumors after one month. No patients died during the ablation treatment. The observed postoperative complications (Table 3) resolved within a week. A total of 142 complications were further classified according to the Clavien–Dindo classification. Grade I complications accounted for 60.6% (*n* = 86), and grade II complications accounted for 39.4% (*n* = 56). Complete ablation in a single session was not achieved in 15 patients. Six of the 96 tumors (6.3%) were incompletely ablated because the tumors were very close to the hepatic arterial and portal venous systems. Patients complained of severe pain and required abandonment. In another 9 cases, the failure was caused by tumor size (4 cases), the tumor abutting the diaphragm (4 cases), and the tumor abutting (distance <5 mm) intrahepatic vessels (1 case). In the 15 failure cases, there were no lesions near the bile duct. One month later, the contrast-enhanced CT scans indicated 9 viable residual tumors in the remaining 90 patients (10%), and a second session of percutaneous ablation was performed. Altogether, complete ablation was achieved in a single session in 84.4% of patients (81 out of 96). This rate was slightly lower than that of common hepatic cancer [8], which can reach a 90–98% complete ablation rate with ordinary percutaneous MWA. Of note, the 5-year OS and 5-year LTP were close to those of common hepatoma [9]. The 90 patients survived a median of 51 months, and the OS rates at 1, 3, and 5 years were 94.2%, 74.3%, and 49.1%, respectively. The 1-, 3-, and 5-year LTP rates were 9.4%, 59.3%, and 72.6%, respectively. In the control group, all patients underwent resection with a median survival of 57 months. There was no difference in median survival (*p* > 0.05). The OS rates of the surgery group at 1, 3, and 5 years were 91.7%, 79.5%, and 56.3%, respectively. There was no significant difference in OS between the two groups (*p* > 0.05).

## 4. Discussion

The morbidity of rare SHC readily increases due to the widespread practice of liver cancer screening among patients with chronic hepatitis B and/or hepatocirrhosis [10, 11]. Compared to other subtypes of liver cancers, for SHC, neither the clinical symptoms nor the medical examination results, such as AFP levels, Child-Pugh classification, tumor-node-metastasis stage, and medical imaging, can specifically differentiate SHC [12, 13]. The definite diagnosis can only be made based on immunohistological staining of the tumor tissue sample. In terms of treatment, surgical excision is the primary choice. Poor surgical candidates have to undergo alternative therapeutics. However, due to the severe tissue fibrosis and necrosis, SHC is very resistant to chemotherapy and transarterial chemoembolization. Thermal ablation can potentially be used to treat SHC. Compared to surgery, thermal ablation has less perioperative mortality and a shorter hospital duration [14]. It has been widely used to treat lung cancer and other types of liver cancers [7]. MWA as one type of thermal ablation can provide a consistently high intratumoral temperature and ablate large volumes with multiple probes; thus, MWA as a suitable therapeutic technique may improve the treatment efficacy in inoperable SHC [15, 16]. However, in clinical use, due to the high puncture resistance, high thermal resistance, and poor thermal conductivity caused by heavy fibrosis and necrosis, the failure rate of MWA in the treatment of SHC is very high. To the best of our knowledge, no study has specifically investigated and optimized thermal ablation to treat SHC.

First, tissue biopsy and histological examination of the lesion are always recommended before ablation. The ablation operator normally is not aware of SHC due to the extremely low morbidity; therefore, it is highly possible to achieve incomplete ablation or complete ablation failure if the operator follows the regular MWA procedure. Once biopsy information is available, a suitable ablation plan can be developed accordingly. In this retrospective study, 96 patients with SHC were treated with a personalized ablation regimen. In brief, the combination of preoperative biopsy and diagnostic imaging assisted the operator in deciding the ablation strategy. Low ablation power and intermittent ablation could significantly improve the tolerance level of patients. The extended ablation time could ensure complete ablation to a certain degree. Second, different types of SHC still require respective ablation strategies. Through the failure cases, we summarize three different types of SHC: (1) if few tumor cells are embedded in an extensive fibrous component, the tumor may be very solid with a hard core, indicating very poor thermal conductivity (Figure 1(a)); (2) cases of mild/moderate levels of fibrous components dispersedly distributed among abundant viable tumor cells normally indicate that the tumor is too rubbery to be effectively punctured (Figure 1(b)); and (3) the mixture of fibrous components and extensive necrotic tumor tissue can form a very rigid tumor capsule that can also prevent puncture (Figure 1(c)). More specifically, if the tumor lesion is less than 10 mm in size, direct puncture followed by ablation is recommended. In other cases, no-touch ablation at 2 abutting sites (tumor size: <15 mm), 4 sites (tumor size: 15–30 mm), and up to 5 sites (tumor size: 30–50 mm) can be performed to completely ablate tumors with at least a 10 mm safety margin. The protocol for NTMSWA we developed demonstrated high therapeutic efficacy that was comparable to surgical resection. In addition, we further compared the therapeutic efficacy of NTMSWA with the ablation efficacy in reported studies. Since there are very limited relevant reports, we only found two papers with regard to thermal ablation of intrahepatic cholangiocarcinoma (ICC). In one study, Kim et al. reported the treatment outcome of 29 ICC lesions in 20 patients with radiofrequency ablation (RFA) [17]. The OS rates at 6 months, 1 year, 2 years, and 4 years were 95%, 70%, 60%, and 21%, respectively. In another study, Fu et al. used RFA to treat ICC and reported that the OS rates at 1 year and 3 years were 87.5% and 37.5%, respectively [18]. Both reports demonstrated the poor prognosis of patients with ICC. By contrast, in this study, bile duct adenocarcinoma and intrahepatic cholangiocarcinoma (ICC) accounted for ∼90% of cases. The OS rates of our patients treated with personalized ablation regimens at 3 and 5 years were 74.3% and 49.1%, respectively. The outcomes are much better than those of patients treated with routine RFA. On the contrary, it further demonstrates that patients with SHC can benefit from the personalized ablation regimen.

Finally, caution is warranted. (1) A limited number of patients were recruited in this study, and the sample size may have led to bias. (2) This was a single-center study, and the results may not be generalizable. (3) It was a retrospective study and thus has all the relevant disadvantages. In future studies, prospective randomized trials with adequate cases will be performed. A thorough comparison of the treatment efficacy and safety between MWA and surgery will be investigated in the treatment of SHC.

In summary, by adopting no-touch, multiple-site, low-power, intermittent ablation, SHC less than 50 mm in size can be completely ablated.

## Figures and Tables

**Table 1:** The average scope of the MWA probe in a freshly excised porcine liver using varying power and time at 37°C. The diameter of the MWA probe is 2 mm.

Power/time	Transverse diameter (mm)	Vertical diameter (mm)	Carbonized width (mm)	Carbonized length (mm)
30 W/3 min	23.8 ± 1.4	34.8 ± 1.7	5.5 ± 0.8	19.0 ± 1.8
30 W/5 min	25.1 ± 1.2	39.0 ± 1.8	6.9 ± 0.6	20.8 ± 2.4
30 W/8 min	26.0 ± 0.9	46.3 ± 2.1	7.1 ± 0.7	24.5 ± 3.3
40 W/3 min	25.5 ± 1.5	36.8 ± 1.3	6.3 ± 0.8	19.6 ± 1.5
40 W/5 min	26.2 ± 1.2	45.0 ± 2.6	7.5 ± 1.3	22.8 ± 2.8
40 W/8 min	37.9 ± 2.1	53.8 ± 2.7	8.1 ± 0.9	26.5 ± 3.6
50 W/3 min	33.8 ± 2.4	39.1 ± 1.5	6.5 ± 0.6	21.0 ± 2.3
50 W/5 min	35.1 ± 1.9	49.2 ± 2.9	7.7 ± 0.7	27.8 ± 3.1
50 W/8 min	46.0 ± 3.5	59.8 ± 3.8	9.2 ± 1.2	31.5 ± 3.5

**Table 2:** Demographics of 137 patients with SHC.

	NTMSWA	Surgery	Comparison *p* value
*Age*			0.4869
<65	72 (75%)	33 (80.5%)	
≥65	24 (25%)	8 (19.5%)	

*Sex*			0.2908
M	33 (34.4%)	18 (33.9%)	
F	63 (65.6%)	23 (56.1%)	

*HV infection*			0.3405
HBV	30 (31.3%)	15 (36.6%)	
HBV + alcohol use	57 (59.4%)	25 (71%)	
HCV	9 (9.4%)	1 (2.4%)	

*Hepatic cirrhosis*			0.3751
Positive	87 (90.6%)	39 (95.1%)	
Negative	9 (9.4%)	2 (4.9%)	

*Tumor size (mm)*	Mean 24.7 mm	Mean 26.5 mm	0.3541
<30	75 (78.1%)	29 (70.7%)	
30–50	21 (21.9%)	12 (29.3%)	

*Tumor location*			0.5426
Right lobe	66 (68.8%)	26 (63.4%)	
Left lobe	30 (31.3%)	15 (36.6%)	

*AFP (μg/L)*			0.8767
≤200	69 (71.9%)	30 (73.2%)	
>200	27 (28.1%)	11 (26.8%)	
ALT (IU/L)	33.5 ± 18.6	29.4 ± 16.5	0.2243
AST (IU/L)	34.2 ± 14.7	31.2 ± 12.7	0.2574
Platelet count (109/L)	132.5 ± 62.5	157 ± 77.2	0.0527

*Total bilirubin (μmol/L)*			0.1324
<34	30 (31.3%)	16 (39.1%)	
34–50	66 (68.8%)	25 (60.9%)	

*Albumin (g/L)*			0.7089
>35	60 (62.5%)	27 (65.9%)	
28–35	36 (37.5%)	14 (34.1%)	

*Child-Pugh score*			<0.001
A	27 (28.1%)	30 (73.2%)	
B	69 (71.9%)	11 (26.8%)	

*BCLC staging*			0.5426
A	30 (31.3%)	15 (36.6%)	
B	66 (68.8&)	26 (63.4%)	

*Histologic type*			0.2219
BDA	60 (62.5%)	21 (51.2%)	
ICC	27 (28.1%)	12 (29.3%)	
HCC	9 (9.4%)	8 (19.5%)	

BDA, bile duct adenocarcinoma; ICC, intrahepatic cholangiocarcinoma; HCC, hepatocellular carcinoma.

**Table 3:** Postoperative complications of 96 patients with sclerosing hepatic carcinoma after CT-guided percutaneous microwave ablation.

	Characteristic	Case
Major	Transient pleural effusion	5
	Transient perihepatic effusion	3
	Liver dysfunction	6

Minor	*Hepatalgia*	
	Mild	30 (31.3%)
	Moderate	15 (15.6%)
	Severe	3 (3.1%)
	*Nausea and vomiting*	21 (21.9%)
	*Anepithymia*	12 (12.5%)
	*Abdominal distension*	9 (9.4%)
	*Fever*	14 (14.6%)
	*Wound Pain*	
	Mild	18 (18.8%)
	Moderate	6 (6.3%)

## Data Availability

The data used to support the findings of this study are available from the corresponding author upon request.
